# Needs and Experiences With Health Care Providers of Adult Rare Disease Patients and Caregivers of People With Rare Diseases: Protocol for a Qualitative Study

**DOI:** 10.2196/53362

**Published:** 2024-04-22

**Authors:** Tina Černe, Eva Turk, Spela Mirosevic, Danica Rotar Pavlič

**Affiliations:** 1 Deparment of Family Medicine Univeristy of Ljubljana Ljubljana Slovenia; 2 Center for Digital Health and Social Innovation Department of Health Sciences Univerisity of St. Pölten St. Pölten Austria; 3 Medical Faculty University of Maribor Maribor Slovenia

**Keywords:** rare diseases, patients, caregivers, needs, barriers, access to health care

## Abstract

**Background:**

Rare diseases in Europe are defined as diseases with a prevalence of less than 5 per 10,000 people. Despite their individual rarity, the total number of rare diseases is considerable. Rare diseases are often chronic and complex, affecting physical, mental, and neurological health. People with rare diseases face challenges such as delayed diagnosis, limited medical support, and financial burden. Caregivers, usually family members, bear significant physical and emotional burdens. Understanding the experiences of patients with rare disease and their caregivers is critical to effective care, but this is still underresearched. Better support and understanding of the challenges faced by both patients and caregivers is clearly needed. Our study will explore the experiences and needs of people with rare diseases and caregivers of people with rare diseases in relation to accessing health services.

**Objective:**

This study aims to explore the experiences of patients with rare disease and their caregivers with Slovenian health care providers and to create a theoretical model of needs and experiences.

**Methods:**

This is a qualitative thematic analysis study, using the codebook approach. The study will conduct semi–open-ended interviews to understand the experiences and needs of people with rare diseases and caregivers of people with rare diseases in relation to accessing health services. The interview questions will be based on an extensive literature review. Data from the interviews will be analyzed using thematic analysis to identify patterns and build a thematic map. Data will be analyzed by at least 2 coders. To ensure reliability, respondent validation will be conducted and negative cases investigated. Any discrepancies will be resolved by consulting the entire research team until a consensus is reached.

**Results:**

This study was not specifically funded. However, author TČ is supported by grant number P3-0339 from the Slovenian Agency for Research and Innovation. This study was approved by the Medical Ethics Committee of the Republic of Slovenia (0120-47/2022/3), and recruitment is expected to begin in May 2024, with data analysis results anticipated by the end of 2025.

**Conclusions:**

This study will fill an important research gap in Slovenia by exploring the needs and experiences of people living with rare diseases and their caregivers. The results will contribute to the broader field of rare diseases and add knowledge that can inform future research processes and intervention strategies. It also aims to identify neglected areas that have a significant impact on the lives of people with rare diseases. This study is important not only because it addresses the immediate needs of the Slovenian rare disease community, but also because it contributes to a discussion on patient-centered care, health policy design, and the inclusion of psychosocial components in health care.

**International Registered Report Identifier (IRRID):**

PRR1-10.2196/53362

## Introduction

### Rare Diseases in Europe and Slovenia

In Europe, rare diseases are defined as diseases with a prevalence of less than 5 per 10,000 people in the population [1]. While individual disease incidence is small, the collective number of all types of rare diseases is large [2]. The estimated number of rare diseases is currently between 6000 and 8000 [3]. They are usually chronic and complex and are associated with physical, mental, or neurological disorders. The psychosocial and emotional impact on affected individuals and families is significant and often exacerbated by a lack of adequate community support and services [4].

### Health Care Needs of Patients With Rare Disease

Rare diseases, due to their multisystemic nature and associated cognitive and developmental challenges, manifest a wide range of symptoms, resulting in diverse health care needs. Managing these symptoms necessitates the involvement of various health care professionals, the use of medical devices, and “orphan drugs” [5]—specialized medicinal products developed to treat, diagnose, or prevent specific rare diseases [6].

### Challenges Faced by Patients With Rare Disease and Caregivers

Rare diseases present numerous challenges, not only for those directly affected but also for their caregivers. These challenges include delayed diagnosis, difficulties accessing health care, and the financial burden of medical treatment [7]. Caregivers of individuals with rare diseases, who are often parents or spouses, shoulder a substantial physical and emotional burden without receiving financial compensation [8]. Their responsibilities encompass providing transportation, running errands, offering emotional support, monitoring symptoms, and performing additional household tasks [9,10]. Many caregivers are forced to reduce or quit their jobs due to these caregiving responsibilities, leading to additional financial strain [11]. The caregivers’ physical and mental well-being directly impacts the level of care they can provide to patients with rare diseases [2].

### Research Gap in Slovenia

In Slovenia, rare diseases pose a significant public health concern and represent a considerable challenge for the health care system [12]. It is roughly estimated that around 150,000 patients are affected by rare diseases in Slovenia [13], which emphasizes the importance of addressing this issue [14]. Recently, a register of rare, nonmalignant diseases was established in the Republic of Slovenia, which should provide valuable insights into this topic. While some studies have been conducted, such as the one by Halec et al [15] on the impact of rare diseases on an individual’s quality of life, and others by Stanimirović et al [12-14] focusing on systems development and strategies to improve care for patients with rare disease, there remains a notable absence of research centered on the experiences and needs of patients with rare disease and their caregivers in Slovenia.

### Addressing Research Gap by This Qualitative Study

In Slovenia, there is little research on the experiences of caregivers of people with rare diseases, especially compared with the studies on caregivers worldwide. While studies often focus on specific diseases and their pathophysiology [8], a comprehensive exploration of the experiences of both patients and caregivers is crucial for health care providers. Such insights can aid in developing treatments and support services that align with the unique challenges faced by this population [16].

Preliminary findings of a scoping review [17] and discussions with representatives of various rare disease associations highlight the pressing need for better support and understanding of such issues and that there is a lack of knowledge in the field about the challenges experienced by caregivers and people with rare diseases. The Ministry of Health states in the work plan in the field of rare diseases in the Republic of Slovenia for the period 2021-2030 that it would be useful to research the needs of patients with rare diseases in Slovenia, with special attention to social aspects, palliative care, psychological support, and economic aspects [18]. There is, therefore, a need to identify clear areas for change to help researchers, health care providers, and health policy makers develop, plan, and facilitate better services. The establishment of the rare non-malignant diseases registry of the Republic of Slovenia [19] and the development of a more comprehensive rare disease ecosystem [14] are important steps, but qualitative research focusing on the experiences of people living with rare diseases will complement these efforts and provide valuable insights.

### Potential Contributions of This Study

This research study holds the potential to contribute significantly to science and the rare disease community in Slovenia by providing a better understanding of the human dimension, which is essential for developing patient-centered policies that consider the psychosocial, emotional, and practical challenges faced by people with rare diseases and their caregivers; identifying gaps in access to health services and psychosocial support; offering insights into priority areas for action that can guide policy makers; and filling a critical research gap in Slovenia by adding valuable knowledge to the rare disease field.

By addressing these aspects, this study has the potential to make a substantial and unique contribution to both the scientific community and the rare disease community in Slovenia.

### Objectives

The primary aim of the study is to explore the needs and experiences of adults with rare diseases and caregivers of people with rare diseases with health care service providers in Slovenia. The secondary aim is to identify gaps in access to health care services and psychosocial support.

The objectives of the research are as follows: (1) to gain insights into the needs and experiences of adults with rare diseases with health care providers in Slovenia; (2) to gain insight into the needs and experiences of caregivers of people with rare diseases with health care service providers in Slovenia; and (3) based on the obtained data, develop a thematic map of experiences and needs.

### Research Questions

Our research questions are as follows: (1) What are the needs and experiences of adults with rare diseases in relation to health care providers in Slovenia? (2) What are the needs and experiences of caregivers of people with rare diseases in relation to health care providers in Slovenia?

## Methods

The reporting of this study follows the guidelines of the Consolidated Criteria for Reporting Qualitative Studies in the areas that can be applied to a protocol [20].

### Research Team and Reflexivity

In this qualitative research study, the interviews will be conducted by TČ, who has a master degree in psychology and is currently working as a researcher. TČ has previous experience with interviews and has spent over a year researching rare diseases and access to health care. Informal discussions were held with representatives of rare disease associations in Slovenia before the study began. These discussions served to develop an initial understanding of the challenges faced by the rare disease community. The main goal was to create a basic knowledge base and gain deeper insights into the problems faced by people living with rare diseases and their caregivers. Participants were informed that the study was part of a doctoral degree pursuit. Disclosure of this information provided transparency regarding the academic aims of the researcher and the context in which the study was conducted. The motivation for the research is rooted in the identified lack of comprehensive studies in this area and emphasizes a commitment to contribute meaningful knowledge to better support people living with rare diseases.

### Study Design—Theoretical Framework

The study uses a conceptual framework based on the biopsychosocial model of health [21], which recognizes the interconnectedness of biological, psychological, and social factors associated with rare diseases [22,23]. The biopsychosocial model requires a multidisciplinary approach [24] and emphasizes the importance of a dynamic and empathetic dyadic relationship between clinicians and patients [25]. This approach includes examining various health-related factors, such as access to psychological support, impact on mental health, social support (family support, self-help groups or networks, and help from caregivers), availability of health care services, and financial resources for health-related needs. By applying the biopsychosocial model, this study aims to provide a comprehensive understanding of the unique experiences of people living with rare diseases and, based on this, make recommendations to improve health care services for people living with rare diseases.

The study follows the approach of qualitative thematic analysis [20]. Given the larger scope of our study and the expected high number of participants, we deliberately chose a “codebook” thematic analysis approach [26]. This structured approach aligns with the pragmatic demands of applied research offering a systematic and organized framework for handling a large volume of qualitative data [26]. We will use a structured coding framework to develop and document the analysis [27]. The codebook developed inductively after (some) data familiarization and coding will serve as a tool to guide data coding and a way of mapping or charting the coded data [26]. Themes that will be developed at an early stage after familiarization, will later be refined or new themes developed based on the subsequent inductive data engagement and analytical process [27].

In the final phase of the analysis, the identified themes generated from the coded data will be used to create a thematic map, and the results will be compared with the existing and new data to assess their relevance and reliability [26].

### Participant Selection

Participants will be selected through purposive and snowball sampling. Participants will be invited by the research team by email or telephone. Email invitations will be sent by the research team using contact details from a public database of the National Contact Point for Rare Diseases, to which individuals have previously given consent to be contacted. We will also ask participants to invite other people with rare diseases and caregivers of people with rare diseases to participate in the study by using snowball sampling.

We will use snowball sampling, as it is difficult to identify and reach people with rare diseases and their caregivers. We will start with an initial group of participants contacted through the National Contact Point for Rare Disease, conduct interviews, and collect data, followed by an explanation of the snowball sampling process. We will ask the initial participants to provide us with their contact details or to facilitate communication with those who are eligible and willing to participate in the study.

In addition, the research team will contact the pediatric clinic and collaborate with the doctors in the Department of Family Medicine by email. The email will include a presentation of the study explaining the purpose, methodology, potential benefits, and the importance of participation in identifying suitable participants, as well as relevant study documents such as an information leaflet, consent forms, and contact details of the research team. These documents will serve as a reference for the pediatric clinic to inform eligible participants about the study. Approval and cooperation from the relevant authorities at the pediatric clinic will be obtained before contact is made. The aim of contacting the pediatric clinic is to invite eligible caregivers of people with rare diseases to participate in the study, and the aim of contacting the Department of Family Medicine is to invite adult patients with rare diseases.

To avoid overrepresentation of a particular rare disease in the sample, we will follow the guideline that for each rare disease included, a maximum of 2 participants (either the adult patient or the caregiver) will be included.

### Sample Size

Braun and Clarke [28] suggest that studies of experiences and needs collected through interviews require a sample size of 15 to 30 people in order to effectively uncover patterns while maintaining a focus on individual experiences.

Considering the concept of information power, which suggests that a broader study aim, a less specific combination of participants for the research question, and the inclusion of cross-case analysis may require a larger sample size, we decided on a target sample size of at least 40 participants [29]. This includes 20 adults with rare diseases and 20 caregivers of people with rare diseases. As Malterud et al [29] suggest, the appraisal of information power will be repeated during the process, supported by an initial analysis. After the first 3 interviews, an initial review of the data will be done and first suggestions of relevant theory will be made [29].

We will also try to identify and document the reasons for nonparticipation, which will contribute to the transparency and reliability of the study.

### Setting

This study is being conducted in Slovenia. It includes health care providers at all 3 levels of health care (primary, secondary, and tertiary care). Data will be collected either in our research offices or participants will have the option of a home visit or digital interview. As caregivers of people with rare diseases may have ongoing care needs [30], we decided to offer them the option of a home visit or digital interview. This gives participants the opportunity to take part at a time and place that is convenient or safe for them [31]. The individual interviews with adults diagnosed with a rare disease will be conducted independently. Caregivers will have the option to participate in the interviews either separately or, if they prefer, together with the person they care for. This flexibility aims to accommodate the preferences and comfort levels of both participants and caregivers.

### Description of Sample

We will include adult patients with rare diseases and caregivers of people with rare diseases in our study. It is important to emphasize that our research will focus on rare, nonmalignant diseases. To determine the rarity of the disease, we will use the European definition of rare diseases, according to which rare diseases are categorized as those that affect less than 5 in 10,000 people in the population. To ensure a comprehensive representation of experiences, we aim to include people with different rare diseases. In addition, our sample will indirectly include people with impaired decision-making capacity, such as those with developmental or cognitive impairments, by involving their caregivers. This decision is in line with ethical considerations, as we recognize that it is difficult for people with developmental disabilities to give fully informed consent due to the nature of their condition. Involving caregivers is seen as an ethical strategy to ensure a full understanding of the research topic while upholding the principles of respect and protection of vulnerable populations. Caregivers can be family members, spouses, unmarried partners, close friends, or other people with direct caring responsibilities. To account for possible regional differences in access to health care and support services, our study will include participants from all 9 health regions of Slovenia. The inclusion criteria extend to people who are directly responsible for people diagnosed with a rare disease.

### Data Collection

Separate interview guides will be used to explore the perspectives of adult patients with rare disease and their caregivers. The interview questions were formulated based on a literature review and will be pilot-tested to ensure clarity and effectiveness in data collection. The questions formulated for the interviews are also based on the biopsychosocial model of health [21]. In line with the biopsychosocial model, our interview questions refer not only to biological factors but also to psychological and social dimensions (how the rare disease affects the participants’ social life and how these conditions influence psychological well-being and social interactions). If subsequent interviews reveal significant topics not covered previously, already interviewed participants may be contacted for additional exploration of those specific areas. Audio recording will be used to capture interviews, ensuring accurate and complete data representation. Participants will be informed about the recording, and explicit consent will be obtained. Field notes will be taken during or after interviews to document contextual information and observations that may enhance the understanding of participants’ experiences. The duration of interviews will be based on the natural flow of the conversation, allowing participants to express themselves fully. Specific timeframes will be tailored to individual preferences and needs. As part of the respondent validation process, we plan to present the results of the study to participants and ask for their feedback and comments. While we will not necessarily provide full transcripts, this approach will ensure that participants have the opportunity to validate the results of the study and contribute to their interpretation.

The sociodemographic questionnaire will collect the following data: age, gender, marital status, education level, employment status, relationship to caregiver (caregiver questionnaire), monthly income, housing situation, rare disease diagnosis, year of diagnosis, duration of symptoms, impact of the disease on daily life, nature of costs associated with the rare disease, frequency of medical visits and difficulties in accessing health care services, and the participants’ support networks.

### Data Analysis

Two researchers will code and compare the data. The coding process will be a collaborative effort to ensure reliability and validity. Themes will be derived through a process. First, a series of initial themes will be developed based on the interview guide and existing literature. Coding will be conducted to provide evidence for these initial themes and to identify additional themes derived from the data. The themes and codes will be refined after the initial coding phase through the development and refinement of a coding template. The final phase of coding will be guided by the final template for theme development. We will use the NVivo program (Lumivero), for coding, editing, and structuring the data. We will also carry out respondent validation, that is, we will present results to the participants and ask them for comments [32]. We will also enrich the data obtained by examining so-called negative cases that deviate from the patterns that otherwise emerge through data saturation [33]. By examining these cases, we will increase the reliability of the research and gain better insight into the strengths and weaknesses of the research [33].

### Eligibility Criteria

The eligibility criteria for this study are listed in Textbox 1.

**The eligibility criteria for this study.**

**Inclusion criteria for adults with a rare disease**

- Aged 18 years or older with a confirmed diagnosis of a rare disease
- Written consent to participate in the study of a person with a rare disease
- Persons who use health services in Slovenia
- Willingness to comply with the study protocol

**Exclusion criteria for adults with a rare disease**

- Inability to meet the requirements of the study (Participants with severe cognitive impairment or intellectual disabilities will be included in the study indirectly through their caregivers. As qualitative research relies on participants’ ability to articulate their experiences, cognitive impairment may limit meaningful participation).
- Unwillingness to meet the demands of the study
- Refusal to fulfill the requirements of the study (refusal to actively participate in the in-depth interviews, to complete a sociodemographic questionnaire, and to consent to data collection)
- Persons who are unable to communicate in Slovene or who are not able to sufficiently understand and express themselves in Slovene will be excluded from the study

**Inclusion criteria for caregivers of people with a rare disease**

- Family caregivers who directly care for a person with a confirmed diagnosis of a rare disease. Family caregivers are usually relatives (often the patient’s parents or spouse) who take on most of the physical and emotional burden of caring for the patient without receiving financial compensation (with the exception of family caregivers who receive payment in the form of social benefits) [34]. They carry out the tasks of monitoring, interpreting, making decisions, taking action, making adjustments, providing care, accessing resources, working with the sick person, and negotiating with the health system [35]
- Written consent to participate in a study of the caregiver of a person with a rare disease

**Exclusion criteria for caregivers of people with a rare disease**

- Unwillingness to comply with the requirements of the study (refusal to actively participate in in-depth interviews, to complete a sociodemographic questionnaire, and to consent to data collection)
- Inability to meet the study requirements (lack of cognitive ability to participate in the study, such as severe cognitive impairment, Alzheimer disease, or intellectual disability)
- Persons who have not reached the legal age of adulthood in Slovenia (younger than 18 years) will be excluded from the study
- Persons with whom it is not possible to communicate in the Slovene language or who are not able to understand and express themselves sufficiently in the Slovene language will be excluded from the survey
- Persons who provide care in the context of an employment relationship and who are not close persons to the person they are caring for (eg, paid home caregivers or home help, persons employed in social care institutions) will be excluded

### Ethical Considerations

The medical ethics committee of the Republic of Slovenia considers the research to be ethically justifiable. The commission notes that all necessary documentation has been included, the statement is written in a way that is understandable for the participants, the security of data storage is guaranteed, and the anonymization of responses is ensured. The document bears the number 0120-47/2022/3. Explicit informed consent will be obtained from all participants. This comprehensive process will ensure participant understanding, adherence to ethical principles, and compliance with the statement approved by the medical ethics committee. The data collected as part of this study will be stored securely in both physical and electronic form, with access to the data restricted to those directly involved in the research. The electronic data will be anonymized by assigning identification codes and stored in a special database located on the premises of the Department of Family Medicine at the Faculty of Medicine in Ljubljana. Before the data are stored in the database, an anonymized version of the research data is created in order to preserve its usefulness while ensuring the confidentiality of the participants. Data protection is ensured by secure passwords, and passwords are stored separately. Participants will not receive any financial or nonmonetary compensation for their participation in this study.

## Results

This study was not specifically funded. However, the author TČ is funded by the Slovenian Agency for Research and Innovation under grant number P3-0339. Data collection through semistructured interviews for the study is scheduled to begin in May 2024 and is expected to be completed on 30 December 2024.

## Discussion

### Principal Results

This study aims to gain a deeper understanding of the needs and experiences of people living with rare diseases and their caregivers when interacting with health care providers in Slovenia and to shed light on the gap between them. The results of the research will be presented in a thematic map of the needs and experiences of people living with rare diseases and their caregivers. The thematic map will serve as a visual representation that will provide an overview of the interconnected themes identified in the analysis and improve our understanding of the complex relationships between the needs and experiences of the target groups. As identified in the exploratory phase of our research, we spoke informally with representatives of rare disease associations in Slovenia to understand the challenges faced by this community. They highlighted key issues, including the need for psychological support, gaps in palliative care for children, concerns about the attitudes of health care providers, and gaps in respite care. In addition, they highlighted issues around delays in diagnosis, poor communication with health care professionals, stigmatization, lack of information, and poorly coordinated care. Ongoing research will shed further light on these issues and contribute to a comprehensive understanding of the gap between needs and available services. We expect to gain a deeper understanding of the barriers to accessing health services, the impact of these barriers on disease progression, and their psychosocial consequences. We expect participants to express their needs in terms of support services and to give examples of good practices that can later be used for better planning of health services. We hope that the results will also point to previously overlooked areas that have a significant impact on the lives of people significantly affected by rare diseases. Based on the results, we will make recommendations to improve health services for people with rare diseases.

### Limitations

A potential challenge of this study arises from the diversity of rare diseases included. Given the uniqueness of each rare disease, it might be difficult to identify commonalities in experiences and needs. While a national register for rare diseases has recently been established in Slovenia, our main resource remains a web-based website that serves as a national contact point for rare diseases. This website lists various associations. Our approach to recruiting participants is a purposive sampling combined with a snowball approach. However, a potential limitation is that individuals who are more involved in supportive communities may be overrepresented in our sample. Consequently, those who are less proactive in seeking the support they need might be underrepresented. To mitigate this bias, we try to use the snowball principle to recruit people who do not belong to these associations and contact participants through doctors. However, it is important to be aware that using the snowball principle may also have a disadvantage. This method could lead to limited diversity among our informants, which could limit the range of perspectives and experiences we can capture [36].

Another challenge is that the study focuses primarily on experiences and needs related to access to health services. However, patients with rare diseases and caregivers of people with rare diseases may face challenges and needs beyond health care, such as education and employment. These aspects may not be fully explored in the study.

### Comparison With Prior Work

Previous research has shown that participants have a significant need for information, particularly in relation to psychological and health care aspects [22,37]. In particular, the Depping study highlights the importance of personalized information and the desire for better access to experts and treatments within the health care system [22].

In line with the aims of our study, we found that caregivers and patients often encountered health care professionals whom they perceived as lacking the necessary knowledge and understanding of them or (in the case of caregivers) the person they were caring for [22,37-40]. Consequently, they saw a noticeable gap in their access to vital and necessary care, emphasizing the urgency of better training for health care providers [22,37-40].

Our study protocol represents a step forward in capturing the specific needs and experiences of people living with rare diseases and their caregivers in Slovenia and differs from previous large-scale surveys, such as the comprehensive study conducted in the United States [40]. In contrast to these large-scale initiatives, our focus is tailored to the Slovenian health care landscape and provides a local understanding of the challenges faced by this population. By focusing on the specific context of Slovenia, our study aims to fill existing gaps and contribute valuable insights to the current state of knowledge in rare disease research.

### Conclusions

This study will fill an important research gap in Slovenia by exploring the needs and experiences of people living with rare diseases and their caregivers. The results will contribute to the broader field of rare diseases and add knowledge that can inform future research processes and intervention strategies. It also aims to identify neglected areas that have a significant impact on the lives of people with rare diseases. This study is important not only because it addresses the immediate needs of the Slovenian rare disease community, but also because it contributes to a discussion on patient-centered care, health policy design, and the inclusion of psychosocial components in health care.

## Data Availability

The data sets generated or analyzed during this study are not publicly available due to confidentiality and ethical considerations but are available from the corresponding author on reasonable request.
